# Synthesis of ternary copper antimony sulfide via solventless thermolysis or aerosol assisted chemical vapour deposition using metal dithiocarbamates

**DOI:** 10.1038/s41598-022-08822-9

**Published:** 2022-04-04

**Authors:** Fadiyah Makin, Firoz Alam, Mark A. Buckingham, David J. Lewis

**Affiliations:** 1 Department of Materials, The University of Manchester, Oxford Road, Manchester, M13 9PL UK; 2 Department of Physics, College of Science, Jazan University, Jazan, 82817 Saudi Arabia; 3 Department of Chemistry, The University of Manchester, Oxford Road, Manchester, M13 9PL UK

**Keywords:** Chemistry, Materials science

## Abstract

Copper antimony sulfide (Cu-Sb-S) has recently been proposed as an attractive alternative photovoltaic material due to the earth-abundant and non-toxic nature of the elements, high absorption coefficients and band gaps commensurate with efficient harvesting of solar photonic flux across multiple phases of Cu-Sb-S. These materials are therefore highly desirable and sustainable and scalable deposition techniques to produce them are of interest. In this paper, we demonstrate two facile, low-temperature and inexpensive techniques (solventless thermolysis and aerosol-assisted chemical vapor deposition (AACVD)) for the preparation of binary digenite (Cu_1.8_S), chalcocite (Cu_2_S) and stibnite (Sb_2_S_3_) and several phases of ternary copper-antimony-sulfide (Cu_2x_Sb_2(1−x)_S_y_, where 0 ≤ x ≤ 1). It was found that by utilising these different techniques and varying the ratio of Cu:Sb, pure phases of ternary chalcostibite (CuSbS_2_), fematinite (Cu_3_SbS_4_) and tetrahedrite (Cu_12_Sb_4_S_13_) can be achieved. Two single-source precursors were investigated for this purpose, namely the diethyldithiocarbamate (DTC) complexes of copper and antimony Cu(DTC)_2_ and Sb(DTC)_3_. These were decomposed both individually (to produce binary materials) and combined (to produce ternary materials) at different ratios. From the solventless thermolysis and AACVD methods, either particulate or thin film material was formed, respectively. These materials were then characterised by powder XRD, SEM, EDX and Raman spectroscopies to determine the crystalline phase, material morphology and uniformity of elemental composition. This analysis demonstrated that as the Cu-content increases, the phase of the ternary material changes from chalcostibite (CuSbS_2_) and fematinite (Cu_3_SbS_4_) at a low Cu:Sb ratio to tetrahedrite (Cu_12_Sb_4_S_13_) at a high Cu:Sb ratio.

## Introduction

The world is currently facing a serious energy crisis from high consumption of non-renewable fossil fuels. Therefore, there is currently a concerted drive towards developing green routes to produce sustainable energy from renewable sources, such as solar energy^1^. Photovoltaic (PV) devices have the potential to be low-cost and sustainable with high solar to electrical energy conversion efficiencies^2,3^. However, this is highly dependent on the abundance and cost of the initial materials and the scalability of the deposition technique.

Numerous metal chalcogenide materials such as copper selenide (CuSe), cadmium selenide (CdSe), copper indium sulfide /selenide (CIS/Se), copper tin telluride (CuSnTe), copper indium gallium sulfide/selenide (CIGS/Se), copper zinc tin sulfide (Cu_2_ZnSnS_4_) (CZTS), and copper iron tin sulfide (Cu_2_FeSnS_4_) have received attention as solar absorbers in photovoltaic cells in recent years^4–9^. Copper antimony sulfide (CuSbS_2_)^10^, copper antimony selenide sulfide (CuSbSe_x_S_2−x_)^11^ and copper germanium selenide (Cu_2_GeSe_3_)^12^ have recently been investigated as both nanoflakes and nanoparticles for electrochemical energy storage. Semiconducting copper and cadmium chalcogenides have played a significant role in developing high-efficiency solar cells. Copper indium gallium selenide (CuInGaSe) and cadmium telluride (CdTe) thin film solar cells have demonstrated efficiencies of 21.5% and 21.7%, respectively^13,14^. However, these materials have several disadvantages, such as low elemental abundance^15^, high cost, and toxicity of elements such as Cd and Te^16^. These disadvantages have significantly hindered the widespread deployment of these materials as photovoltaic devices.

Ternary copper antimony sulfide has recently been proposed as an alternative PV material, due to the low-cost, earth-abundant, and non-toxic nature of the constituent elements^17^. Ternary copper antimony sulfide also yields high absorption coefficients (over 10^5^ cm^−1^) in several phases^18^, with a suitable band gap for photovoltaic applications^10,19^. However, there are several distinct phases of copper antimony sulfide, (Cu-Sb-S) including Cu_3_SbS_3_ (skinnerite), CuSbS_2_ (chalcostibite), C_12_Sb_4_S_13_ (tetrahedrite), and Cu_3_SbS_4_ (fematinite)^20^. These materials all possess band gap energies between 1.1 and 1.9 eV, with high absorption coefficients (> 10^5^ cm^−1^)^18^, and are thus excellent candidates for solar energy harvesting^21–25^. However, phase-pure material of Cu-Sb-S (such as tetrahedrite) is difficult to achieve and has previously been reported as requiring several weeks and high temperatures for various melting and annealing steps^18,26,27^. Chalcostibite (CuSbS_2_) in particular has been proposed as a candidate for the replacement of CuInS_2_, due to equivalent optical properties and the natural abundance of antimony when compared to indium^18^. Tetrahedrite (Cu_12_Sb_4_S_13_) is another important phase as this material is not only a good candidate for solar absorbance in photovoltaic devices^18^, but also as a candidate for thermal energy harvesting as a thermoelectric device via the Seebeck effect^18^.

The use of single-source precursors for the preparation of binary and ternary metal chalcogenides is of growing importance^18,28–31^. This method is potentially beneficial because of the simplicity, high purity, and tunable size of the produced materials^32^. Copper and antimony dithiocarbamate (DTC) complexes are frequently used for binary and ternary metal sulfide thin film preparation due to their ease of synthesis, clean decomposition at low temperature, and solubility of these complexes in many organic solvents^28, 33–35^. Ternary copper-antimony sulfide nanocrystals have been previously prepared using single-source precursors of antimony diethyldithiocarbamate (Sb(DTC)_3_) and copper diethyldithiocarbamate (Cu(DTC)_2_)^36^. The produced copper antimony sulfide nanocrystals exhibit different compositions, band gaps, and morphologies including rhombic Cu_3_SbS_3_, rectangular CuSbS_2_ nanosheets, and trigonal–pyramidal Cu_12_Sb_4_S_13_ nanocrystals, which have shown promising potential for photovoltaic applications^37^. Zou *et. al.*, synthesized chalcostibite CuSbS_2_ nanocrystals using a colloidal route with dodecanethiol as ligands. A direct band gap energy of 1.59 eV was reported, and they were further demonstrated to having utility in photovoltaic applications^38^. Regulacio *et. al.* used copper and antimony diethyldithiocarbamate precursors for the synthesis of high-purity tetrahedrite (Cu_12_Sb_4_S_13_) nanostructures using solvothermal decomposition^39^.

Numerous methods have been developed for the deposition of particulate, nanoscale and thin film copper antimony sulfides, such as the solvothermal^40^, chemical bath deposition^41^, spin coating^42^, spray pyrolysis^43^, thermal evaporation^44^, and pulsed laser ablation^45^. Solventless thermolysis involves decomposition of a precursor under thermal stress. This method is inexpensive and is inherently more sustainable that most other methods due to the absence of solvent^46^. Solventless thermolysis also produces high yields for the synthesis of desired materials^18^. Different morphologies of metal chalcogenides can be prepared using this method such as nanorods^47^, nanodisks^48^, and cubic nanoparticles^49,50^. However, difficulty in controlling the size of particles in the presence of capping agents has limited progress in designing these nanomaterials^51^.

In the present study, two facile, scalable, and cost-effective methods for the preparation of phase pure binary Sb_2_S_3_, Cu_2_S and Cu_1.8_S are reported, and three distinct phases of ternary copper antimony sulfide (CuSbS_2_, Cu_3_SbS_4_, and Cu_12_Sb_4_S_13_) can be prepared as either particulates (using solventless thermolysis) or thin films (using AACVD). These materials are prepared from single-source precursors of copper and antimony diethyldithiocarbamates (Cu(DTC)_2_ and Sb(DTC)_3_) mixed at different molar ratios of Cu:Sb. The work presented here reports two simple and scalable routes towards several phases of ternary Cu-Sb-S, which are highly relevant for both photovoltaic and thermoelectric applications.

## Experimental

### Chemicals

All chemicals were purchased from Sigma Aldrich and used without further purification, unless specified. Sodium diethyldithiocarbamate trihydrate ((C_2_H_5_)_2_NCS_2_Na·3H_2_O, ≥ 99.0%), antimony trichloride (SbCl_3,_ ≥ 99.5%), anhydrous copper(II) chloride (CuCl_2_ ≥ 98.0%), methanol (CH_3_OH, ≥ 99.8%), absolute ethanol (CH_3_CH_2_OH, ≥ 99.5%), chloroform (CHCl_3_, ≥ 99%) and isopropyl alcohol ((CH_3_)_2_CHOH, ≥ 99.7%). Synthesis of the precursors was performed in air and no special handling or inert atmosphere was required.

### Instrumentation

Elemental analysis (EA) was performed in the micro-analytical laboratory of the University of Manchester. Thermogravimetric analysis (TGA) was conducted using a Perkin Elmer TGA 4000, in a temperature range of 30 to 600 °C and a ramp rate of 10 °C min^−1^ under a nitrogen atmosphere. Powder XRD patterns were recorded using an XRD5-PANaytical X-Pert Pro theta-theta diffractometer (435 mm diameter). The data was collected using detector scans at a grazing incidence angle of 3° and were undertaken with a scan range of 5° to 85° with a 0.03° step at 8 s step^−1^. Scanning electron microscopy (SEM) imaging was performed using an FEI Quanta 650 Field Emission GUN (FEG)-SEM equipped with an Oxford Instruments Xmax 50 Silicon Drift Detector (for EDX microanalysis) with an accelerating voltage of 10—20 kV. Raman spectra were recorded using a Renishaw 1000 Micro- Raman spectrometer, equipped with a 50 × objective, with argon ion laser (514.5 nm, 25% powder).

### Synthesis

#### Copper diethyldithiocarbamate, Cu(DTC)_2_ (1)

**Figure 1:**
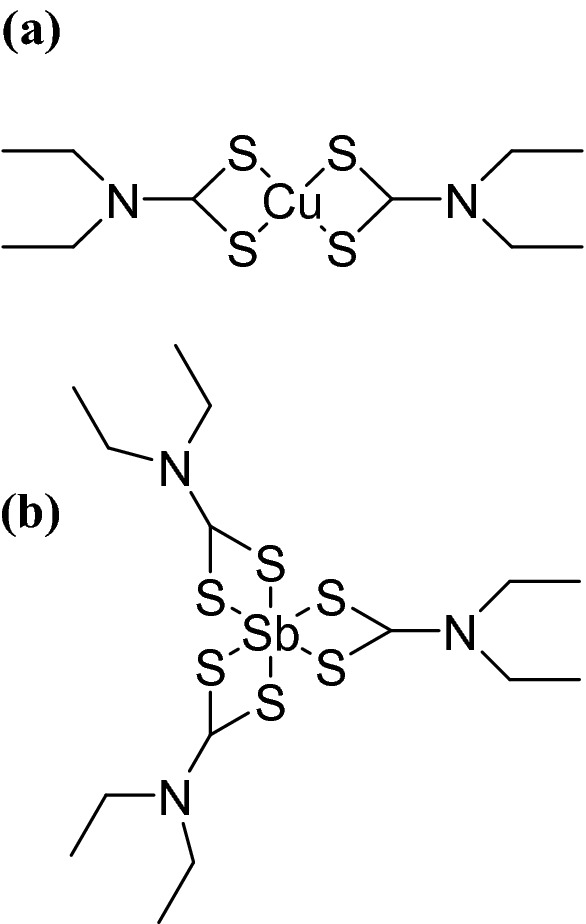
Figure showing the chemical structures of the two single-source precursors utilised in this report. These are (**a**) copper(II) diethyldithiocarbamate (Cu(DTC)_2_) and (**b**) antimony(III) diethyldithiocarbamate (Sb(DTC)_3_).

The synthesis of copper diethyldithiocarbamate (Cu(DTC)_2_) was achieved by adapting a previously reported procedure^37^. Sodium diethyldithiocarbamate trihydrate (10 g, 44 mmol) was dissolved in 100 mL of methanol and stirred until fully dissolved. A separate solution of copper(II) chloride (2.89 g, 22 mmol) was prepared in 25 mL of methanol. The copper solution was then added dropwise to the diethyldithiocarbamate solution under constant stirring for 30 min. A black precipitate was formed, which was filtered, and washed three times with room temperature deionized water and methanol and dried overnight under atmospheric laboratory conditions. Yield 74%. Anal. found: C, 32.90%; H, 5.52%; N, 7.69%; S, 30.05%. Calc.: C, 33.37%; H, 5.61%; N, 7.79%; S, 35.56%. This could be due to some reduction of the Cu(II) to Cu(I), in line with previous reports^28,52^ and our observations of chalcocite (Cu_2_S, Fig. 8) and digenite (Cu_1.8_S, Fig. 4). The chemical structure of the precursor is illustrated in Fig. 1a.

#### Antimony(III) diethyldithiocarbamate, Sb(DTC)_3_ (2)

The synthesis of antimony diethyldithiocarbamate (Sb(DTC)_3_) was achieved by adapting a previously reported procedure^37^. Sodium diethyldithiocarbamate trihydrate (6.76 g, 30 mmol) was dissolved in 100 mL of methanol and stirred until fully dissolved. In a separate solution, antimony (III) chloride (2.26 g, 10 mmol) was dissolved in 25 mL of methanol. The prepared antimony chloride solution was then added dropwise to the diethyldithiocarbamate solution under constant stirring. Once fully added, the reaction mixture was further stirred for 30 min at room temperature. After this time, a yellow precipitate was filtered and washed several times with deionized water and methanol at room temperature. The product (yellow solid) was dried overnight at room temperature in atmospheric laboratory conditions. Yield 86%. Anal. found: C, 31.35%; H, 5.19%; N, 7.25%; S, 33.22%. Calc: C, 31.81%; H, 5.34%; N, 7.42%; S, 33.90%. The chemical structure of the precursor is illustrated in Fig. 1b.

### Preparation of binary Cu_x_S_y_ and Sb_2_S_3_ powders by solventless thermolysis

Solventless thermolysis was used for the synthesis of particulate Cu_x_S_y_ and Sb_2_S_3_ using the single-source precursors Cu(DTC)_2_ and Sb(DTC)_3_, respectively. A measured amount (0.55 mmol) of precursor was placed into a ceramic boat inside a tube furnace and annealed at either 300, 400, 425, 450, or 475 °C for 1 h, under a nitrogen atmosphere. The obtained black product was cooled at room temperature and collected for characterization.

### Preparation of copper-antimony-sulfide (Cu-Sb-S) powders by solventless thermolysis

Copper-antimony sulfide Cu_2x_Sb_2(1−x)_S_y_ was prepared by solventless thermolysis using a mixture of both precursors (Cu(DTC)_2_ and Sb(DTC)_3_) at different molar ratios, where x = either 0, 0.2, 0.4, 0.6, 0.8 or 1 (the respective mol fractions, and absolute input amount (in mmol) are displayed in Table S1). The mixture was dissolved in 10 mL of chloroform to form a homogeneous solution. The solvent was then evaporated under kneading using a pestle and mortar. The obtained mixture was then placed into a ceramic boat and heated in a tube furnace at 450 °C for 1 h under a nitrogen atmosphere. The obtained black product was cooled at room temperature and collected for characterization.

### Preparation of copper-antimony-sulfide (Cu-Sb-S) thin films by AACVD

Prior to deposition, glass slides of the dimensions 1 cm × 2.5 cm were thoroughly cleaned by consecutively washing with deionized water (DI) and acetone for 10 min each, under ultrasonic conditions. The glass slides were then dried in the oven before loading inside a reactor tube for deposition. The deposition of copper antimony sulfide thin films was carried out using the aerosol-assisted chemical vapor deposition (AACVD) technique. The different molar fractions of Cu(DTC)_2_ and Sb(DTC)_3_ were dissolved in 20 mL of tetrahydrofuran (THF) and transferred to a two-necked round bottom flask. The flask outlet was connected to the reactor tube via rubber tubing, and argon gas was flowed into the inlet. The reactor tube was placed in a carbolite tube furnace and heated to 450 °C for one hour. The aerosol was generated by placing the reaction flask that contained the precursor solution on an ultrasonic aerosol generator. The argon flow (160–180 sccm) transported the aerosol to the hot wall reaction chamber where thermal decomposition took place to form a thin film on the substrates. The films obtained were then characterized by p-XRD, EDX, SEM, Raman, and UV–Vis spectroscopies.

## Results and discussion

### Solventless thermolysis of Cu(DTC)_2_ and Sb(DTC)_3_ to generate binary Cu_x_S_y_ and Sb_2_S_3_ systems

The aim of this research is to generate target phases of Cu-Sb-S substrates through simple, cost-effective routes. Namely, investigating both solventless thermolysis and AACVD deposition techniques to decompose single-source precursors, producing both binary and ternary metal chalcogenides. Initially, the two single-source precursors (Cu(DTC)_2_ and Sb(DTC)_3_) were investigated individually for their thermal decomposition behaviour.

Thermogravimetric analysis (TGA) on the two single source precursors demonstrated that both these precursors decompose in the temperature range 240–340 °C. The Cu(DTC)_2_ precursor observed a single, major decomposition with no further loss in mass with increasing temperature. The Sb(DTC)_3_ precursor also observed a single step decomposition, but was followed by a more gradual decomposition at higher temperature, which is attributed to further loss of elemental sulfur^53^. The TGA results are shown in Fig. S1 and indicates that the two precursors decompose to their respective metal sulfides within a similar temperature range, this makes them ideal candidates to explore further to produce particulate binary metal chalcogenides.

Therefore, thermal decomposition (via solvent-less thermolysis) of the two precursors was performed to generate the respective binary particulate metal chalcogenide material. This was undertaken at a range of temperatures for 1 h. The crystallinity of the resultant particulate material was analysed by powder X-ray diffraction (p-XRD). The resultant p-XRD patterns of the particulate material generated from Sb(DTC)_3_ at 300, 400, 425, 450, and 475 °C are shown in Fig. S2. This analysis revealed that the produced material was crystalline and corresponded to orthorhombic stibnite (Sb_2_S_3_, ICDD: 01-073-0393)^54^ with lattice parameters a = 11.313 Å, b = 3.833 Å, and c = 11.225 Å.

Analysis of the particulate product of the Cu(DTC)_2_ precursor, decomposed at 300, 350, 400, and 450 °C was also undertaken. The resulting p-XRD diffraction patterns of these particulate materials are shown in Fig. S3. Again, this analysis also showed that the resulting material was crystalline, with the major diffraction peaks corresponding to rhombohedral digenite (Cu_1.8_S, ICDD 00-023-0962)^55^ with minor peaks in the pattern corresponding to impurities of monoclinic chalcocite (Cu_2_S, ICDD 00-033-0490, indicated by a * on the p-XRD spectra, Fig. S3)^56^, which is particularly prevalent for reactions performed at lower temperatures. At higher decomposition temperatures (450 °C), there is no observable Cu_2_S present, producing phase pure Cu_1.8_S. The observed lattice parameters are a = 3.930 Å, b = 3.930 Å, and c = 48.650 Å, with the major diffraction peaks corresponding to the (015), (018), (101), (110), planes (as shown on Fig. S3). The morphology and elemental composition of these binary Cu_1.8_S and Sb_2_S_3_ particulate materials were further investigated by SEM and EDX analysis, and are discussed later, together with the ternary Cu_2x_Sb_2(1−x)_S_y_ systems.

### Synthesis of ternary Cu_2x_Sb_2(1−x)_S_y_ (0 ≤ ***x*** ≤ 1) powder by solventless thermolysis

Following the success of producing particulate binary Cu_1.8_S and Sb_2_S_3_ from their respective single-source precursors, the precursors were then investigated for their ability to synthesise the more complex ternary metal sulfides. The TGA analysis demonstrated that both precursors decompose to their corresponding metal sulfides in a similar temperature range. Both precursors are soluble in chloroform, toluene, and other organic solvents. Therefore, we concluded that these complexes are good candidates for decomposition in tandem to generate ternary metal sulfides^29^, at lower temperatures and a faster time than typically employed for this synthesis (650 °C^26^ and 3 weeks^27^).

These two precursors were therefore investigated towards the preparation of ternary copper-antimony-sulfide (Cu-Sb-S) material at various molar ratios of Cu:Sb. The temperature selected for this investigation was 450 °C, as this temperature produced the phase purest material in the Cu(DTC)_2_ decomposition (Fig. S3). Therefore, solutions containing both precursors (Cu(DTC)_2_ and Sb(DTC)_3_) of various ratios (Table S1) were used to generate particulate Cu_2x_Sb_2(1−x)_S_y_ material, where the mol fraction (x) used was 0 (*i.e.* only Sb_2_S_3_), 0.2, 0.4, 0.6, 0.8 and 1 (*i.e.* only Cu_1.8_S).

### SEM and EDX analysis of particulate Cu_1.8_S, Sb_2_S_3_, and Cu_2x_Sb_2(1−x)_S_y_ generated by solventless thermolysis

**Figure 2:**
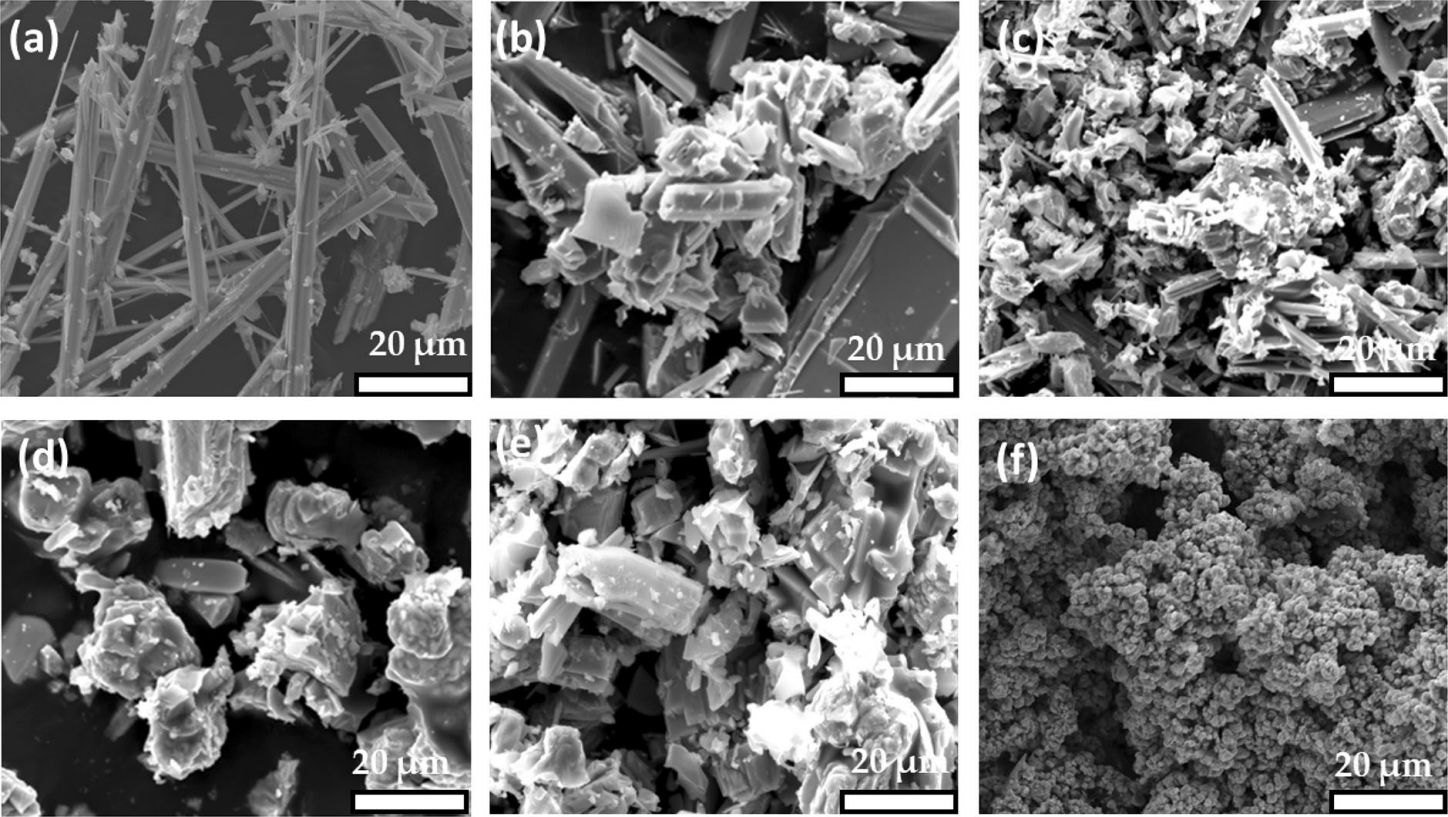
Representative SEM images of particulate Cu_2x_Sb_2(1−x)_S_y_ prepared by solventless thermolysis at 450 °C for 1 h where x = (**a**) 0, (**b**) 0.2, (**c**) 0.4, (**d**) 0.6, (**e**) 0.8 and (**f**) 1. These were recorded at 15 k eV using secondary electron SEM.

Initially, SEM and EDX spectroscopies were used to study the morphology and elemental composition of the generated particulate Cu_2x_Sb_2(1−x)_S_y_ material, at various values of x. Representative SEM images of the six Cu_2x_Sb_2(1−x)_S_y_ systems, where x = 0, 0.2, 0.4, 0.6, 0.8 and 1 are shown in Fig. 2. SEM analysis of pure Sb_2_S_3_ (x = 0) revealed micro-rod structures (Fig. 2a), which are consistent with previously reported particulate Sb_2_S_3_, prepared using hydrothermal decomposition of an antimony diethyldithiocarbamate precursor^34^. As the Cu content of the systems increased, the morphology changed to a mixture of shorter-length micro-rods and agglomerated clusters at x = 0.2, as shown in Fig. 2b. As the Cu content increased to x = 0.4, again both rods and clusters are present, as shown in Fig. 2c. Increasing the molar fraction of Cu to x = 0.6 and x = 0.8 showed no rod-character and only cluster-character (Fig. 2d and e). Finally, the morphology of pure Cu_1.8_S, where x = 1, showed even further (and smaller scale) clustering, resembling a floret-like morphology (Fig. 2f).

These six particulate samples were then assessed for elemental composition by EDX spectroscopy. The Cu_2x_Sb_2(1−x)_S_y_ systems where x = 0.2, 0.4, 0.6, and 0.8 showed the characteristic peaks of copper, antimony, and sulfur (Fig. S4). Figure S5 shows both the expected elemental composition and the observed experimental composition for both Cu and Sb, based on the EDX analysis, the data is also tabulated in Table S2. As shown in Fig. S5, the linear relationship between the expected and observed Cu and Sb mol% of the various synthesised particulate Cu_2x_Sb_2(1−x)_S_y_ systems are in good agreement, with both observing a > 98% fit). The one outlier is the Cu and Sb composition in the x = 0.6 system, which is deficient in Cu and rich in Sb (Table S2).

### EDX spectroscopic mapping of Cu_2x_Sb_2(1−x)_S_y_

**Figure 3:**
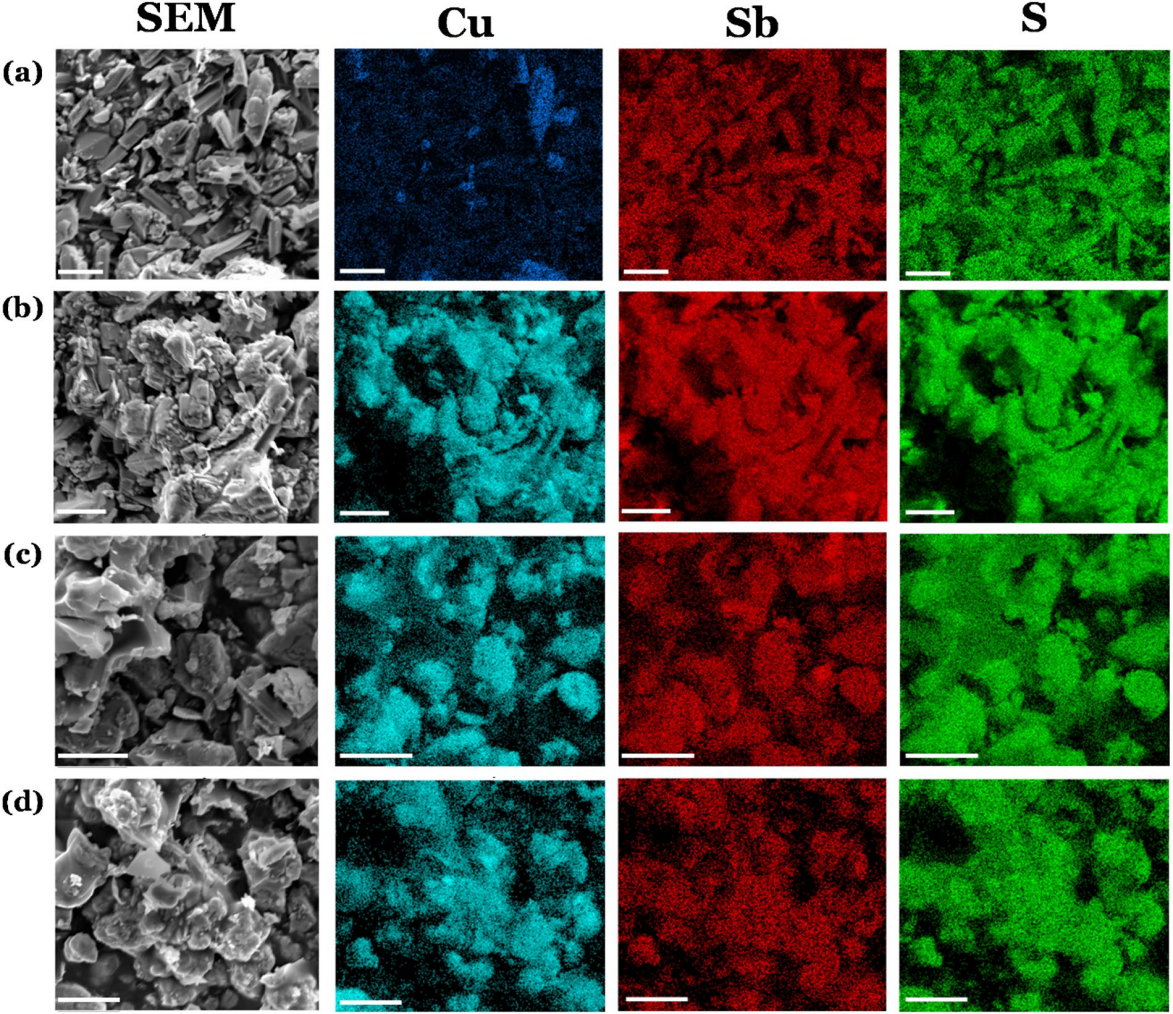
EDX elemental mapping analysis of the various particulate Cu_2x_Sb_2(1−x)_S_y_ systems prepared by solventless thermolysis at 450 °C for 1 h, where x = (**a**) 0.2, (**b**) 0.4, (**c**) 0.6 and (**d**) 0.8. Elemental mapping is also shown for (green) sulfur (Kα), (blue) copper (Kα) and (red) antimony (Lα). These were recorded at 15 k eV using secondary electron SEM. All scale bars presented correspond to 10 µm.

EDX elemental mapping was performed on the four Cu_2x_Sb_2(1−x)_S_y_ systems where 0 < x < 1. This was not performed where x = either 0 or 1, as pXRD analysis above already demonstrated no contamination of Sb in the x = 1 system or Cu in the x = 0 system. The resultant element maps are shown in Fig. 3, which show the spatial distribution of Cu, Sb and S. From this analysis it is clear that the Cu and Sb are predominantly co-localised, which suggests formation of the ternary metal sulfide material, as opposed to two separate phases of Cu_x_S_y_ and Sb_x_S_y_. However, we do note apparent Cu-rich areas in the material where x = 0.2 (Fig. 3a) which suggests some limited phase separation. Despite this, there is strong evidence of the presence of alloyed Cu_2x_Sb_2(1−x)_S_y_.

This analysis yields useful insight into the generated particulate Cu_2x_Sb_2(1−x)_S_y_ material, but does not indicate whether these structures are crystalline, or the phase or absolute composition of these particulate systems. To investigate this further, powder X-ray diffraction was employed on all six synthesised Cu_2x_Sb_2(1−x)_S_y_ systems.

### Powder X-ray diffraction analysis of Cu_2x_Sb_2(1−x)_S_y_

With the SEM analysis showing a change in morphology with increasing Cu character in the particulate Cu_2x_Sb_2(1−x)_S_y_ systems, and the EDX elemental mapping analysis showing the presence of both Cu and Sb localised together, these six particulate systems were investigated by powder XRD to determine both the crystallinity and the phase of the various Cu_2x_Sb_2(1−x)_S_y_ systems produced.

**Figure 4:**
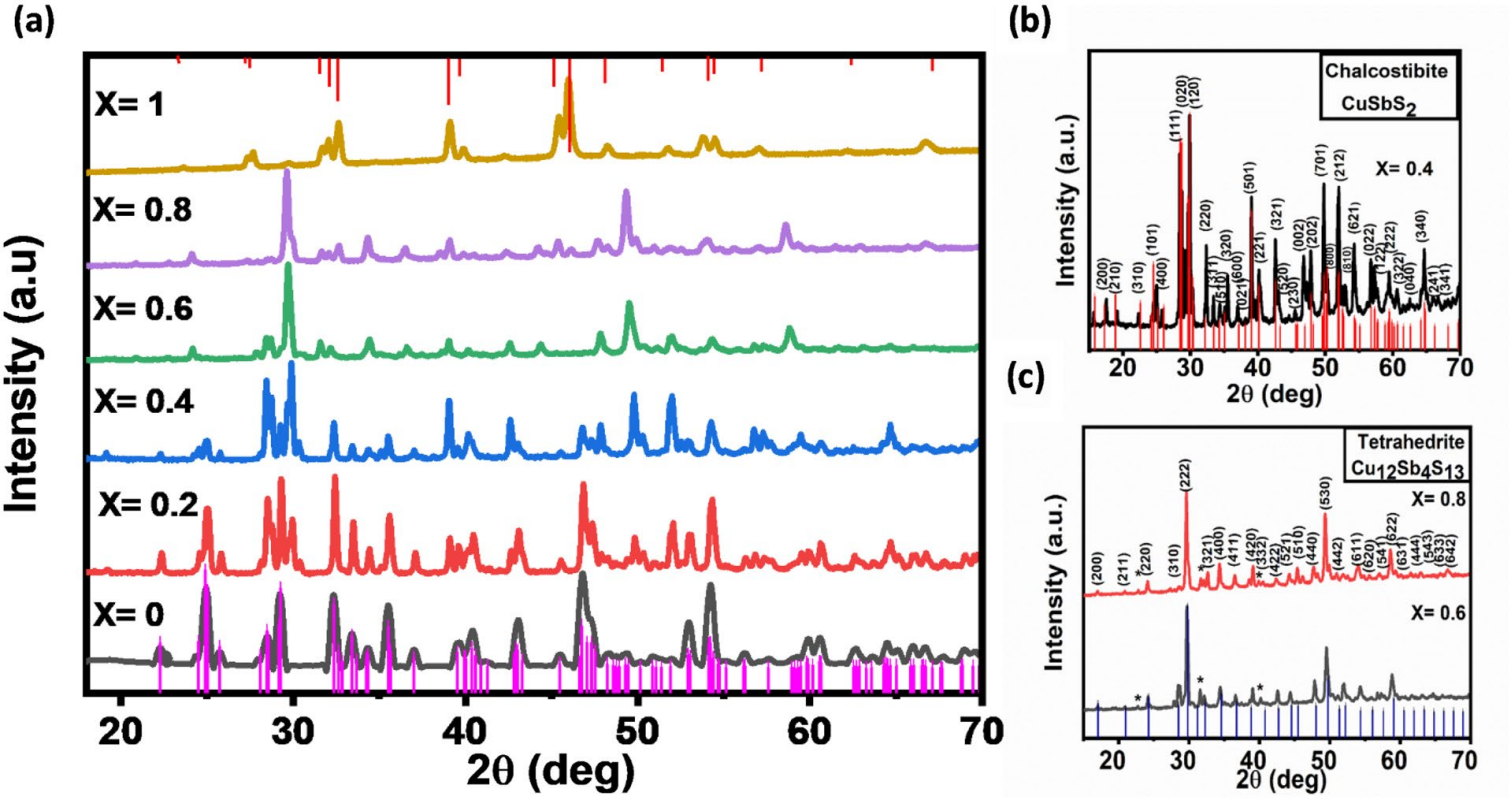
Plots showing the p-XRD analysis of Cu_2x_Sb_2(1−x)_S_y_ produced by solventless thermolysis at 450 °C, where the mole fraction of Cu:Sb (x) was altered as in (**a**) x = 0, 0.2, 0.4, 0.6, 0.8 and 1, where x = 0 produced pure Sb_2_S_3_ (ICDD: 01-073-0393, stick pattern at bottom) and x = 1 produced pure Cu_1.8_S (ICDD 00-023-0962, stick pattern at top). Also shown are the individual p-XRD patterns for (**b**) x = 0.4, showing pure chalcostibite (CuSbS_2_, ICDD: 00-044-1417) and (**c**) x = 0.6 and 0.8, showing pure tetrahedrite Cu_12_Sb_4_S_13_ (ICDD: 01-074-0270).

The p-XRD spectra of the six particulate Cu_2x_Sb_2(1−x)_S_y_ systems are shown in Fig. 4. As previously discussed, the XRD pattern for the sample where x = 0 could be indexed to orthorhombic stibnite (Sb_2_S_3_), and the XRD pattern for x = 1 could be indexed to rhombohedral digenite (Cu_1.8_S). Next, the samples where 0 < x < 1 were analysed. For the sample where x = 0.2, XRD showed peaks consistent with ternary chalcostibite (CuSbS_2_), with impurities of Sb_2_S_3_. Increasing the mole fraction to x = 0.4 resulted in the formation of orthorhombic chalcostibite (CuSbS_2_, ICDD: 00-044-1417) (Fig. 4b). Upon further increasing the molar ratio of Cu to x = 0.6, a different phase of ternary copper antimony sulfide was observed; tetrahedrite (Cu_12_Sb_4_S_13_, ICDD: 01-074-0270), with lattice parameters of a = 11.423 Å, b = 14.174 Å*,* and c = 3.765 Å, with some impurities of Cu_1.8_S. Previous reports have demonstrated that it is difficult to avoid a small number of impurities in the synthesis of tetrahedrite^57,58^, particularly when using copper and antimony xanthate (dithiocarbonate) precursors^18^. The presence of these impurities also account for the slight discrepancy in the EDX elemental composition analysis. Finally, increasing the molar ratio of Cu to x = 0.8 resulted in the generation of predominately pure phase cubic tetrahedrite (Cu_12_Sb_4_S_3_, ICDD: 01-074-0270), as shown by the XRD pattern in Fig. 4c.

### Raman Spectroscopy of the Cu_2x_Sb_2(1−x)_S_y_ systems

**Figure 5:**
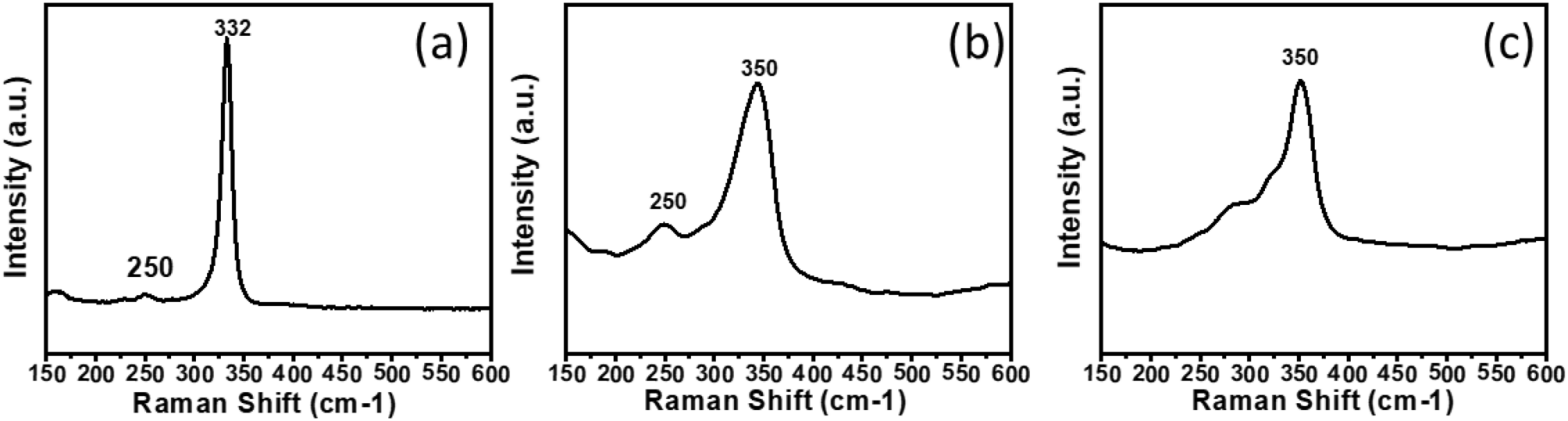
Raman spectra of the particulate Cu_2x_Sb_2(1−x)_S_y_ systems where x = (**a**) 0.4, (**b**) 0.6 and (**c**) 0.8. Indicating the presence of chalostibite (CuSbS_2_) and tetrahedrite (Cu_12_Sb_4_S_13_), respectively.

Raman spectroscopy was used to further study the particulate binary Cu_1.8_S and Sb_2_S_3_ and ternary Cu_2x_Sb_2(1−x)_S_y_ systems (as shown in Fig. 5). The three systems where x = 0, 0.2 and 1 are shown in Fig. S6. For the system where x = 0.4, Raman spectroscopy further confirms the presence of pure chalcostibite (CuSbS_2_), with a sharp peak at 332 cm^−1^, and lower intensity peak at 250 cm^−1^ ^18^. The p-XRD analysis indicates that the x = 0.6 and 0.8 systems were both tetrahedrite, of varying purities (Fig. 4). The Raman spectra of these systems displayed a sharp peak at 350 cm^−1^, corresponding to cubic tetrahedrite Cu_12_Sb_4_S_13_^18^. In the x = 0.6 system, a further peak at 250 cm^−1^ is also observed, which corresponds to Cu–S bonding (from the Cu_1.8_S impurity)^59–61^. This peak reduces in intensity as the mol fraction of Cu increases. These results correlate well with those previously reported for chalcostibite and tetrahedrite, obtained by solventless thermolysis of xanthate precursors of Cu and Sb^18^.

### AACVD of Cu_2x_Sb_2(1−x)_S_y_ (0 ≤ ***x*** ≤ 1) thin film systems

Having successfully synthesised particulate material of binary Cu_1.8_S and Sb_2_S_3_, and ternary CuSbS_2_ and Cu_12_Sb_4_S_13_, by simply altering the ratio of Cu: Sb precursors using solventless thermolysis, we next set out to produce thin films of these materials. For this, aerosol-assisted chemical vapor deposition (AACVD) was utilised. AACVD is a ubiquitous and widely employed method of generating films of metal chalcogenide materials from both metal dithiocarbamate and metal xanthate single-source precursors^28,62–64^. This technique was therefore utilised here to deposit both binary and ternary metal sulfides.

Deposition of Sb_2_S_3_, Cu_9_S_5_ and Cu_2x_Sb_2(1−x)_S_y_ by AACVD onto glass substrates was performed using a method described previously^65^. The aerosol was generated by an ultrasonic humidifier and transported by flowing argon. Deposition of the films was carried out for 1 h at 450 °C, which generated matte black films with good surface coverage, as in Fig. S7. Initially, the morphology and elemental composition analysis was conducted on these six sample films by SEM and EDX spectroscopy.

### SEM, EDX and Elemental mapping analysis of the Cu_2x_Sb_2(1−x)_S_y_ thin films

**Figure 6:**
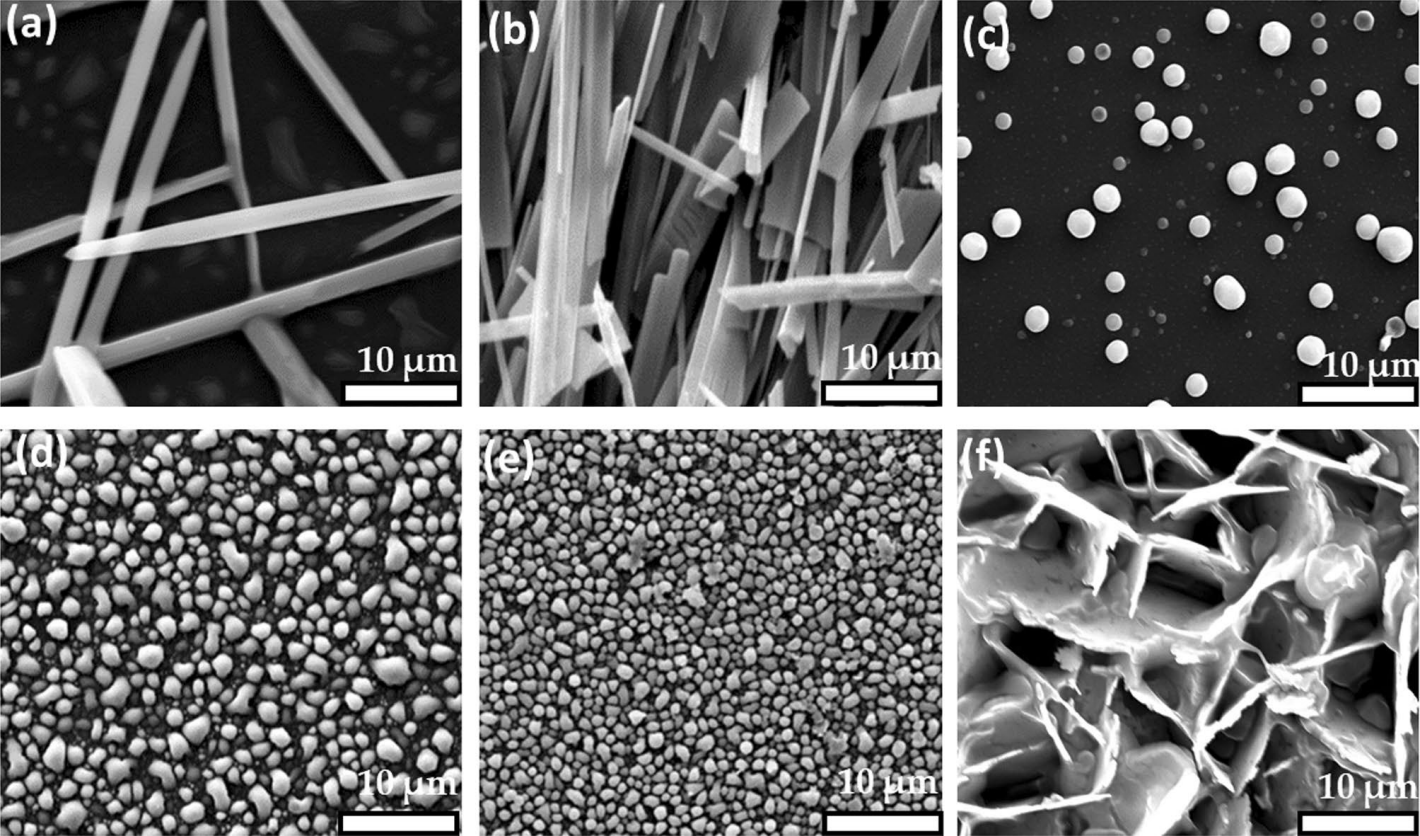
Representative SEM images of Cu_2x_Sb_2(1−x)_S_y_ thin film synthesised by the hot-wall AACVD at 450 °C using various values of x, where x = (**a**) 0, (**b**) 0.2, (**c**) 0.4, (**d**) 0.6, (**e**) 0.8 and (**f**) 1. These were recorded at 15 k eV using secondary electron SEM.

The surface morphology of binary and ternary copper antimony sulfide thin films were initially investigated by SEM. Figure 6 shows representative SEM images of the six investigated films. When x = 0 (*i.e.* when only Sb is present), and x = 0.2, the SEM images again show rod-shaped structures (Fig. 6a,b). Upon increasing x to 0.4, the morphology changed to spherical particles rather than rods, which are also observed upon further increasing x to 0.6 and 0.8 (Fig. 6c–e). Chalapathi *et. al.* has previously reported that thin films of Cu_3_SbS_4_, generated by chemical bath deposition have a spherical crystal morphology^66^. Van Ebden *et. al.* has also reported spherical nanoparticles of tetrahedrite (Cu_12_Sb_4_S_13_)^23^. Therefore, our morphological analysis is in some agreement with the prior literature, despite being different from the particulate material generated by solventless thermolysis (Fig. 2). Finally, when x = 1 (*i.e.* only Cu is present), flake-like sheets were observed (Fig. 6f), which is consistent with a previous report of AACVD decomposition of a bis[N,N-dipropyl-N-(1-naphthoyl)thioureato]copper(II) precursor^67^.

Following the morphological investigation of the thin film Cu_2x_Sb_2(1−x)_S_y_ samples, elemental composition analysis was then investigated through EDX spectrscopy and is summarised in Table S3 (spectra are shown in Fig. S8). For the binary systems where x = 0 only Sb and S were present in the expected ratio (*ca.* 45:55), and x = 1 only Cu and S were present, also in the expected ratio (*ca.* 65:35).

For the ternary systems where 0 < x < 1, a composition of both Sb and Cu were present, alongside S. The ternary systems where x = 0.4, 0.6 and 0.8 all have the expected composition (Table S3). The system where x = 0.2 was found to be Sb-rich and S-deficient, which could again be due to the presence of impurities. Comparison of the EDX analysed expected *vs* observed Cu and Sb content again found a linear correlation (Fig. S9), equal to the particulate Cu_2x_Sb_2(1−x)_S_y_ samples generated by solventless thermolysis. We note a linear correlation between the observed Cu and Sb to that expected when considering the phases of the Cu-Sb-S produced and identified by pXRD (vide infra).

**Figure 7:**
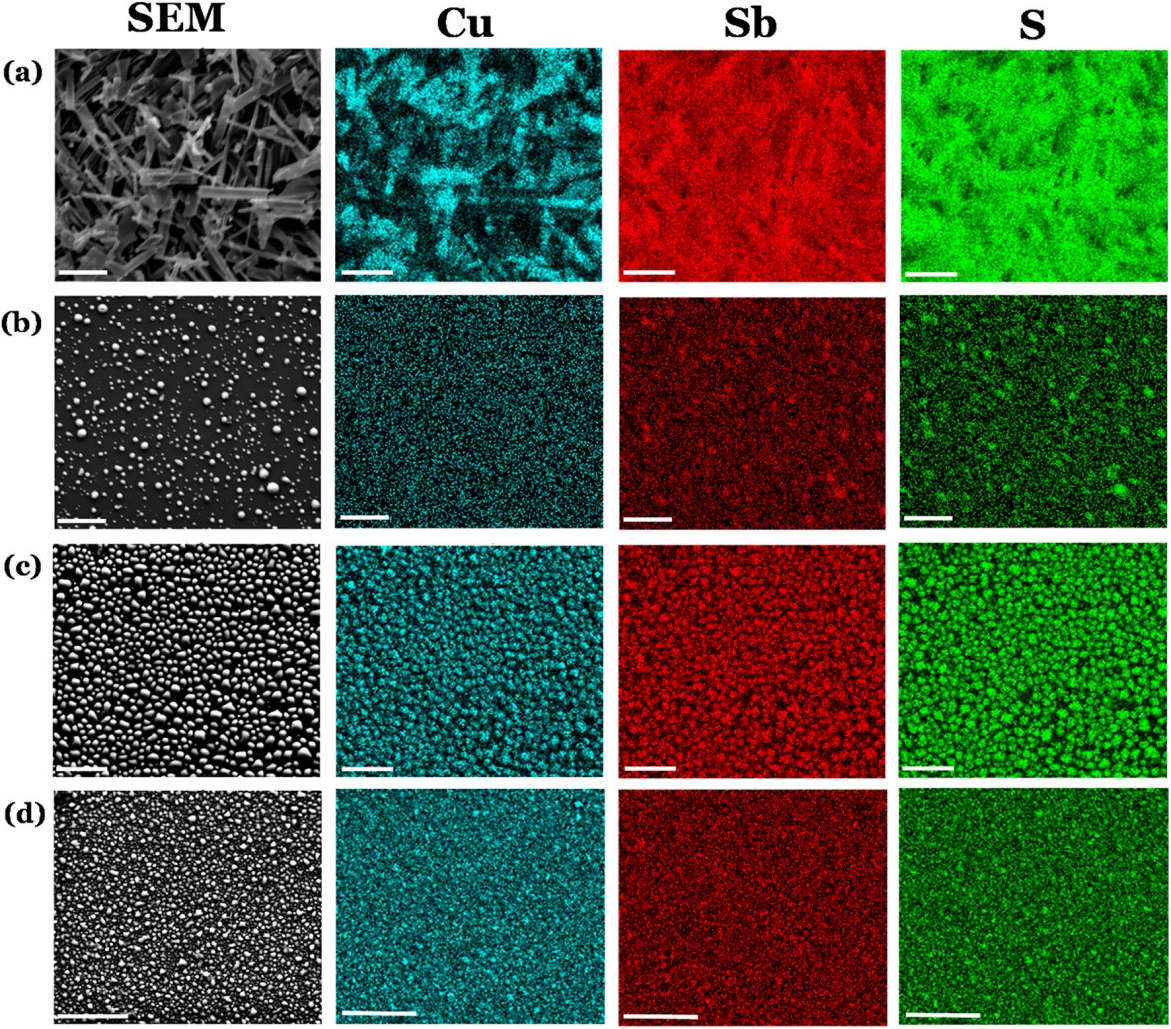
Figure showing both SEM (column 1) and elemental analysis maps (column 2, 3 and 4) for the four Cu_2x_Sb_2(1−x)_S_y_ systems where (**a**) x = 0.2, (**b**) x = 0.4, (**c**) x = 0.6 and (**d**) x = 0.8. These films were deposited by AACVD and decomposed at 450 °C. Elemental mapping is also shown for (green) sulfur (Kα), (blue) copper (Kα) and (red) antimony (Lα). These were recorded at 15 k eV using secondary electron SEM. Scale bars correspond to 10 µm.

**Figure 8:**
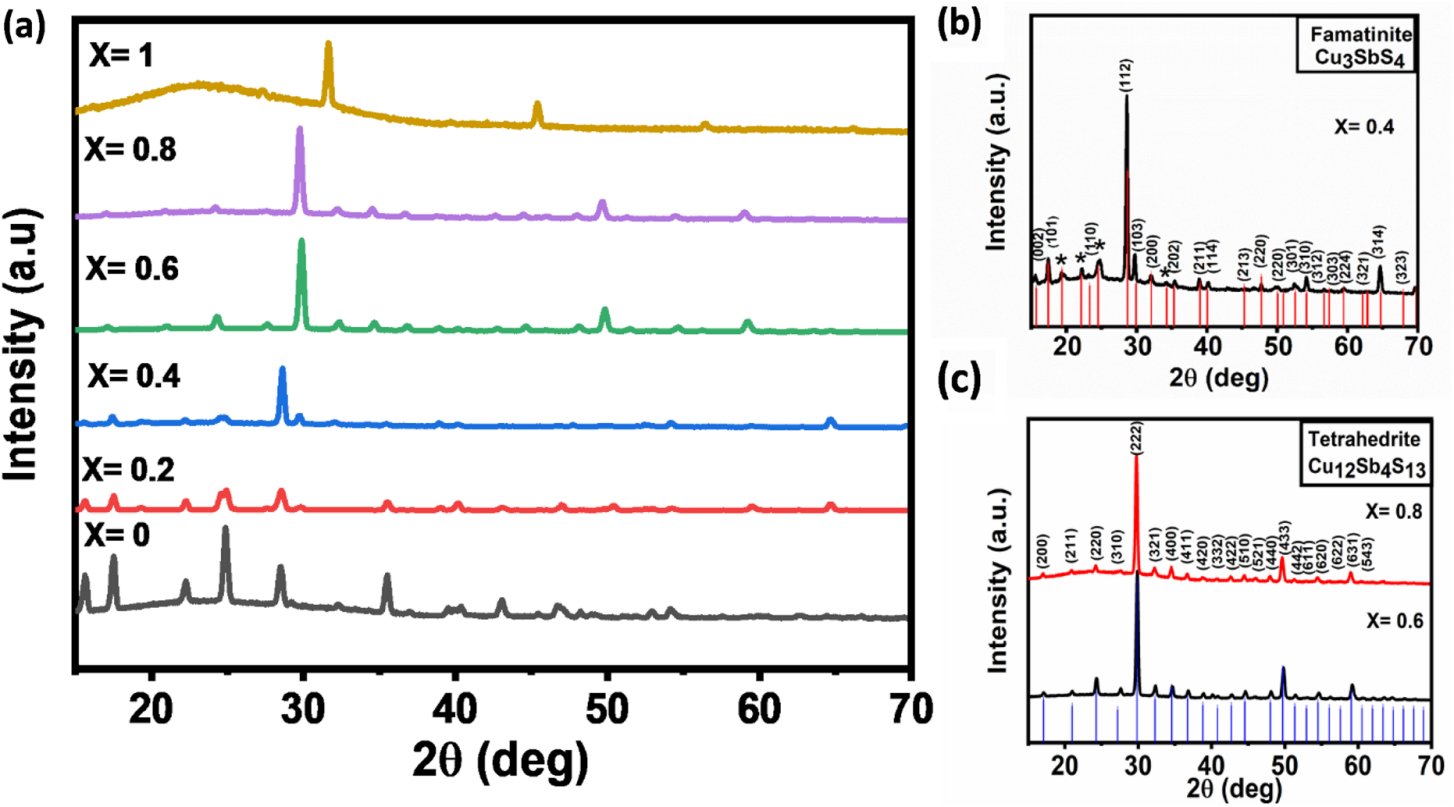
Plots showing the powder-XRD patterns for (**a**) all deposited Cu_2x_Sb_2(1−x)_S_y_ thin films of x = 0, 0.2, 0.4, 0.6, 0.8 and 1. Also shown are the individual patterns for (**b**) x = 0.4 fematinite (Cu_3_SbS_4_, ICDD: 01-071-0555) and (**c**) x = 0.6 and x = 0.8 tetrahedrite (Cu_12_Sb_4_S_13_, ICDD:01-088-0283). These spectra were recorded on the AACVD thin films, deposited at 450 °C.

Having investigated the surface morphology of the deposited thin films, the Cu_2x_Sb_2(1−x)_S_y_ thin films were investigated using EDX spectroscopic elemental mapping on the samples where 0 < x < 1. This analysis visualizes the distribution of all constituent elements (Cu, Sb and S) within the films (Fig. 7). The materials deposited by AACVD demonstrate some Cu-rich areas in the film where x = 0.2 (Fig. 7a). As can also be observed, these Cu-rich areas are also Sb-deficient relative the rest of the material. This analysis indicates that the two binary Cu–S and Sb-S phases were deposited separately, rather than depositing any ternary Cu-Sb-S material. This outcome is supported by the p-XRD analysis (vide infra) which could not accurately determine any distinct ternary phase. In the x = 0.4 thin film, there also appears to be Sb-rich areas (the larger particles in Fig. 7b). This is due to the presence of Sb_2_S_3_ impurities as also shown by the pXRD pattern (Fig. 8).

### Powder XRD analysis of the Cu_2x_Sb_2(1−x)_S_y_ thin films

The SEM, EDX spectroscopy and elemental mapping of the various Cu_2x_Sb_2(1−x)_S_y_ thin films showed that the Cu and Sb are on the whole spatially localised. However, the morphology of these materials was different to those obtained through solventless thermolysis, therefore the thin films were further investigated through powder XRD, to determine the phase of each material.

All deposited thin films where x < 1 displayed sharp peaks in their p-XRD patterns, indicating some crystallinity throughout the film. The deposited film where x = 0 (*i.e.* Sb only) could again be indexed to orthorhombic Sb_2_S_3_ (ICDD: 01-071-2432) with respective lattice parameters of a = 11.2902 b = 3.6953 Å and c = 11.1398 Å; α = β = γ = 90°, which is equivalent to the antimony sulfide generated through solventless thermolysis. As the concentration of Cu increased at x = 0.2, the p-XRD pattern remained that of orthorhombic Sb_2_S_3_, with some further peaks at higher 2θ, which could not be attributed to any products. At x = 0.4, a majority of tetragonal fematinite (Cu_3_SbS_4_, ICDD: 01-071-0555) is observed, with lattice parameters a = 5.3740 Å, b = 5.37540 Å & c = 10.5340 Å (Fig. 8b). Impurities that we attribute to Sb_2_S_3_ are also observed and are marked by an * in Fig. 8b. Notably, this is a different phase of copper-antimony-sulfide to that obtained from the solventless thermolysis deposition at the same mole ratio of Cu:Sb. These different phases in the x = 0.2 and 0.4 systems deposited by either AACVD to solventless thermolysis was compared to the phase diagram for Cu-Sb-S at 500 °C^68^. It has been reported that the thermodynamic product at the two Cu:Sb:S ratios where x = 0.2 and 0.4 (assuming 100% reduction of Cu(ii) to Cu(i)) are chalcosibite (CuSbS_2_). Therefore, the formation of stibnite (Sb_2_S_3_) and fematinite (Cu_3_SbS_4_) demonstrates that AACVD deposition can form kinetic products. We note that solventless thermolysis forms the thermodynamically favourable digenite (C_1.8_S) and AACVD deposits the kinetic chalcocite (Cu_2_S)^68^.

At x = 0.6 and 0.8, the phase produced is cubic tetrahedrite (Cu_12_Sb_4_S_13_ ICDD: 01-088-0283) with lattice parameters a = b = c = 10.2853 Å (Fig. 8c), which is consistent with the Cu_2x_Sb_2(1−x)_S_y_ material obtained from solventless thermolysis. The XRD pattern of the x = 1 thin film has sharp peaks. These peaks can be indexed to cubic Cu_2_S (ICDD: 01-084-1770) with lattice parameters equal to a = b = c = 5.4539 Å. This is again different to the equivalent solventless thermolysis deposition which produced Cu_1.8_S, perhaps indicating less sulfur loss is observed using AACVD and that AACVD in this case is prone to deposit a kinetic, rather than the thermodynamically favourable phase^68^.

### Raman spectroscopy of the Cu_2x_Sb_2(1−x)_S_y_ thin films

**Figure 9:**
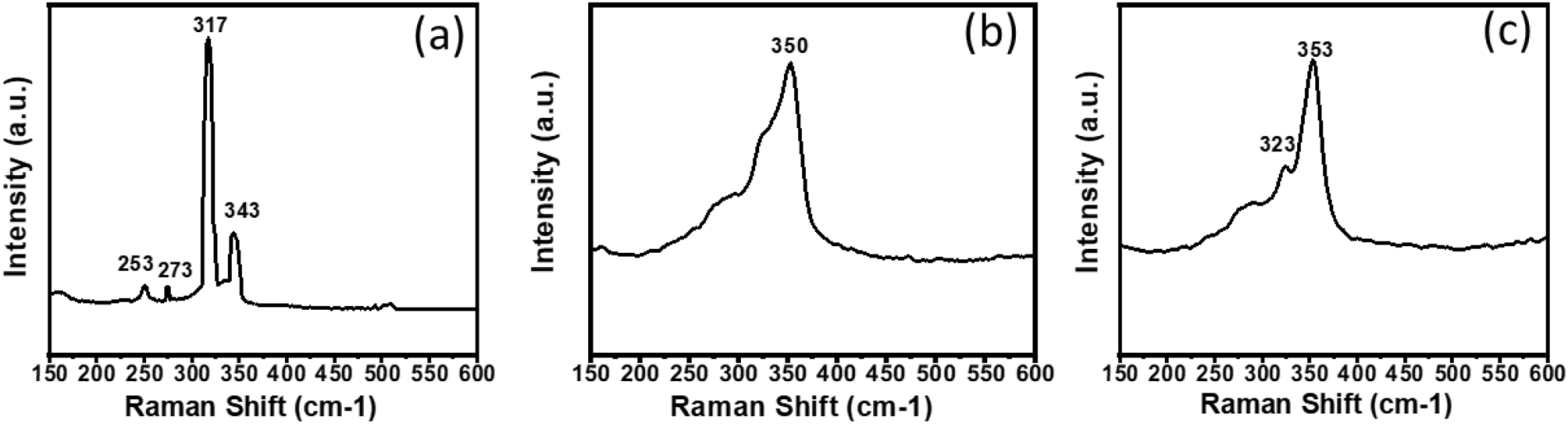
Raman spectra recorded for the AACVD-deposited thin films of Cu_2x_Sb_2(1−x)_ S_y_ where x = (**a**) 0.4 (**b**) 0.6 and (**c**) 0.8. Indicating the presence of (**a**) famatinite (Cu_3_SbS_4_) and (**b**) and (**c**) tetrahedrite (Cu_12_Sb_4_S_13_).

Following the successful characterisation of the phases of the AACVD deposited Cu_2x_Sb_2(1−x)_S_y_ thin films, the films were analysed by Raman spectroscopy. The Raman spectra for the films where x = 0, 0.2 and 1 are shown in Fig. S10. For the thin film deposited when x = 0.4, the peaks in the Raman spectrum appear at 253, 273, 317 and 343 cm^−1^ (Fig. 9a), which correspond to tetragonal famatinite Cu_3_SbS_4_^69^, confirming the p-XRD characterisation. As x was increased to 0.6 and 0.8, the Raman peak was shifted to 352 cm^−1^, which correspond to cubic tetrahedrite Cu_12_Sb_4_S_13_^59,60^ (Fig. 9b). A further peak at 323 cm^−1^ was observed in the x = 0.8 thin film, also indicating pure cubic tetrahedrite Cu_12_Sb_4_S_13_ (Fig. 9c). These results are agreement with the previous literature investigating various phases of copper-antimony-sulfur^18^.

## Conclusions

In this study, binary and ternary Cu-Sb-S phases were produced by the thermal decomposition of both copper and antimony diethyldithiocarbamate single source precursors. We produced particulates and thin films through solventless thermolysis, or aerosol-assisted chemical vapor deposition, respectively. Utilising different methods of deposition resulted in the generation of different phases when altering the ratio of Cu:Sb precursors. Under solventless thermolysis using a low Cu ratio produced chalcostibite (CuSbS_2_), and digenite (Cu_1.8_S) with no Sb precursor present. Using AACVD to deposit thin films resulted in the formation of fematinite (Cu_3_SbS_4_) under a low Cu ratio and chalcocite (Cu_2_S) with no Sb precursor present. Both methods were found to produce stibnite (Sb_2_S_3_) with no Cu present and tetrahedrite (Cu_12_Sb_4_S_13_) with a high ratio of Cu:Sb.

The morphology of these produced materials was characterised by SEM and the elemental composition of these materials was determined through EDX analysis and EDX-mapping. These analyses demonstrated that the morphology of the materials change from a rod-like structure at low Cu content to more particulate material with increasing Cu content. Elemental mapping demonstrated that Cu and Sb were present in the same local environments, indicating the successful synthesis of ternary metal sulfides, which was confirmed by powder XRD and Raman spectroscopy.

This report demonstrates that both deposition methods (solventless thermolysis and AACVD) provide a facile, low-temperature and efficient synthetic approach towards the preparation of phase pure binary and ternary metal sulfides from single-source diethyldithiocarbamate precursors. The synthesised phase-pure ternary copper antimony sulfide materials CuSbS_2_, Cu_3_SbS_4_ and Cu_12_Sb_4_S_13_ are all targets relevant for photovoltaic applications, and it is likely this approach is universal towards the synthesis of ternary metal chalcogenides.

## Supplementary Information

Supplementary Information.
