# Supplementary material for: Synthesis of ternary copper antimony sulfide via solventless thermolysis or aerosol assisted chemical vapour deposition using metal dithiocarbamates
Source: Sci Rep. 2022 Apr 4;12:5627. doi: 10.1038/s41598-022-08822-9 (PMC8979952; doi:10.1038/s41598-022-08822-9)
Supplement: Supplementary file 1 — Supplementary Information. [file 41598_2022_8822_MOESM1_ESM.pdf]

# Supplementary information for:

# Synthesis of ternary copper antimony sulfide *via* solventless thermolysis or aerosol assisted chemical vapour deposition using metal dithiocarbamates

Fadiyah Makin,[a,b] Firoz Alam,[c] Mark A. Buckingham[a] and David J. Lewis[a]*

[a] Department of Materials, The University of Manchester, Oxford Road, Manchester, M13 9PL, UK.

[b] Jazan University, College of Science, Department of Physics, Jazan, 82817, Saudi Arabia.

[c] Department of Chemistry, The University of Manchester, Oxford Road, Manchester, M13 9PL, UK.

*Corresponding author: david.lewis-4@manchester.ac.uk

# Contents:

**Table S1:** Composition used for synthesis of $Cu_{2x}Sb_{2(1-x)}S_y$ powders and thin films

**Figure S1:** Thermogravimetric analysis of the $Cu(DTC)_2$ and $Sb(DTC)_3$ precursors.

**Figure S2:** P-XRD patterns of particulate $Sb(DTC)_3$

**Figure S3:** P-XRD patterns of particulate $Cu(DTC)_2$

**Figure S4:** The EDX spectra of $Cu_{2x}Sb_{2(1-x)}S_y$ samples prepared by solventless thermolysis

**Figure S5:** The amount of copper expected and the amount of copper in $Cu_{2x}Sb_{2(1-x)}S_y$ systems synthesized by solventless thermolysis.

**Figure S6:** Raman spectra of the particulate $Cu_{2x}Sb_{2(1-x)}S_y$ systems where x = (a) 0,(b) 0.2 and (c)1.

**Figure S7:** Photographs of the thin film $Cu_{2x}Sb_{2(1-x)}S_y$ systems deposited by AACVD

**Figure S8:** The EDX spectra of $Cu_{2x}Sb_{2(1-x)}S_y$ thin films prepared by AACVD.

**Figure S9:** The amount of copper expected and the amount of copper in $Cu_{2x}Sb_{2(1-x)}S_y$ thin films synthesized by the AACVD method.

**Figure S10:** Raman spectra recorded for the AACVD-deposited thin films of $Cu_{2x}Sb_{2(1-x)}S_y$ where x = (a) 0 (b) 0.2 and (c) 1.

**Table of data of various mole fraction Cu : Sb precursors used in the synthesis of ternary $Cu_{2x}Sb_{2(1-x)}S_y$**

**Table S1.** Composition used for the synthesis of $Cu_{2x}Sb_{2(1-x)}S_y$ system by the solventless thermolysis and AACVD methods.

| Mole fraction [Cu]/[Cu]+[Sb] | $Cu[DTC]_2$ | $Sb[DTC]_3$ |
|---|---|---|
| 0 | 0 mmol | 0.55 mmol |
| 0.2 | 0.55 mmol | 2.2 mmol |
| 0.4 | 0.55 mmol | 0.80 mmol |
| 0.6 | 0.55 mmol | 0.35 mmol |
| 0.8 | 0.55 mmol | 0.13 mmol |
| 1 | 0.55 mmol | 0 mmol |

## TGA analysis of the individual $Cu(DTC)_2$ and $Sb(DTC)_3$ precursors

The final residual mass of the $Cu(DTC)_2$ precursor was observed to be 13%, which is significantly less than the expected value of 22 % for $Cu_2S$, or 20 % for $Cu_9S_5$. In this case no further loss was observed at higher temperature (Figure S1). The $Sb(DTC)_3$ precursor also observed a single, rapid decomposition, followed by a more gradual decomposition resulting in a product *ca.* 25% of the initial mass, which is also significantly lower than the expected 30% for $Sb_2S_3$. Beyond 425 $^0$C, a gradual loss of further mass is observed, which can be attributed to further loss of elemental sulfur.[1] The TGA results indicate that the two precursors decompose to their respective metal sulfides within a similar temperature range, this makes them ideal candidates to explore further to produce particulate binary metal chalcogenides.

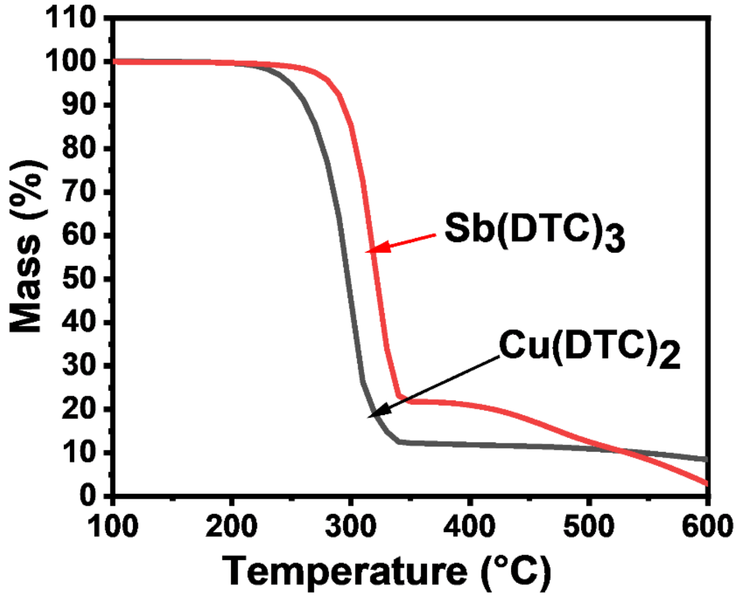


**Figure S1**. Plot showing the TGA decomposition analysis of the $Cu(DTC)_2$ and $Sb(DTC)_3$ and precursors.

## Powder XRD diffraction patterns for solventless decomposition of $Sb(DTC)_3$ at various temperature

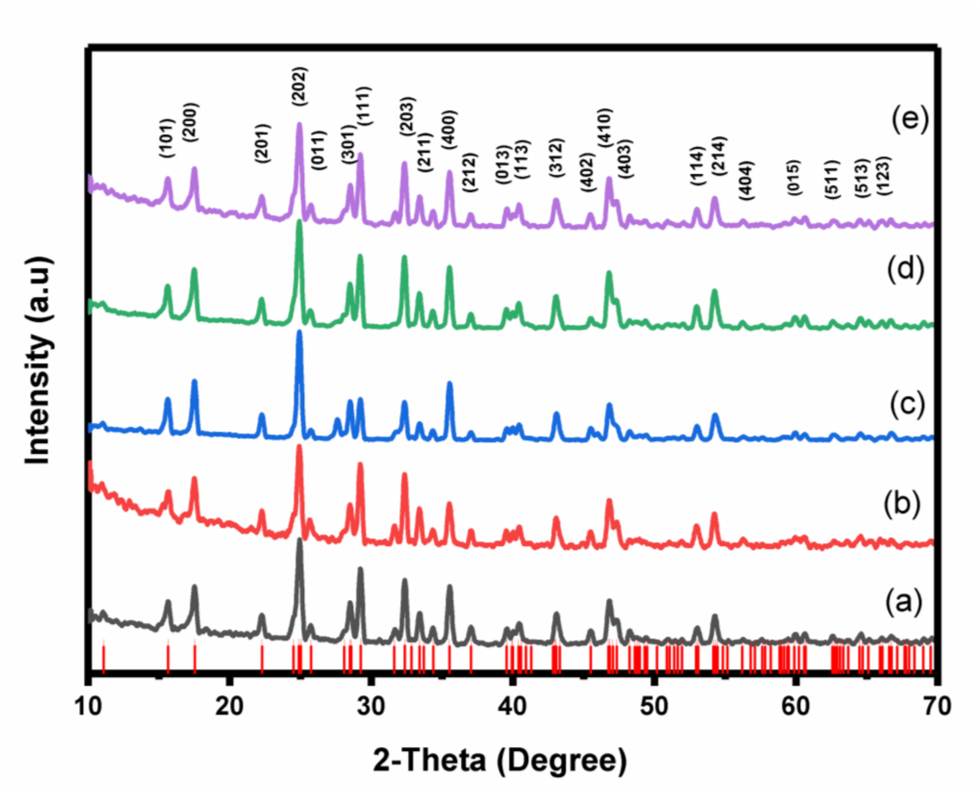


**Figure S2.** Plot showing the p-XRD patterns of particulate $Sb(DTC)_3$ decomposition products, where the decomposition temperature was (a) 300 ˚C, (b) 400 ˚C, (c) 425 ˚C, (d) 450 ˚C and (e) 475 ˚C, decomposed by solvent-less thermolysis for 1 h. All peaks corresponded to standard pattern of stibnite $Sb_2S_3$ (ICDD 01-073-0393)

**Powder XRD diffraction patterns for solventless decomposition of $Cu(DTC)_2$ at various temperature**

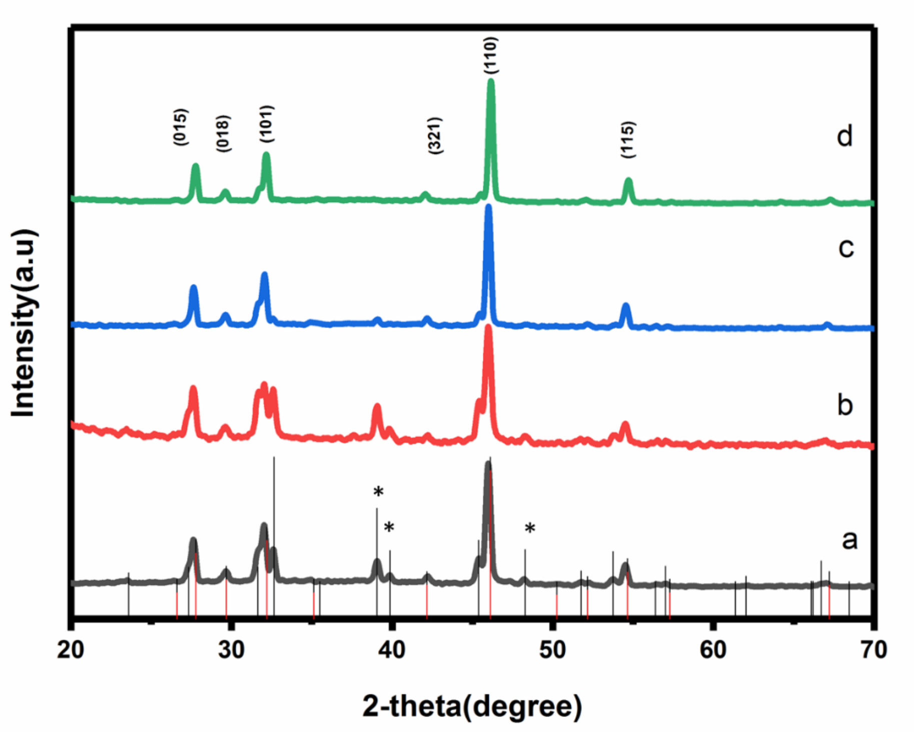


**Figure S3.** Plot showing the p-XRD patterns of particulate $Cu(DTC)_2$ decomposition products, at decomposition temperatures of (a) 300 °C, (b) 350 °C (c) 400 °C and (d) 450 °C, decomposed by solvent-less thermolysis for 1 h. Peaks correspond to digenite ($Cu_9S_5$) ICDD 00-023-0962. Minor phase monoclinic chalcocite ($Cu_2S$) ICDD 01-072-1071 have been marked with an * in part (a), which are still present in (b) and (c).

**Elemental analysis spectra of the particulate $Cu_{2x}Sb_{2(1-x)}S_y$ samples produced by solventless thermolysis**

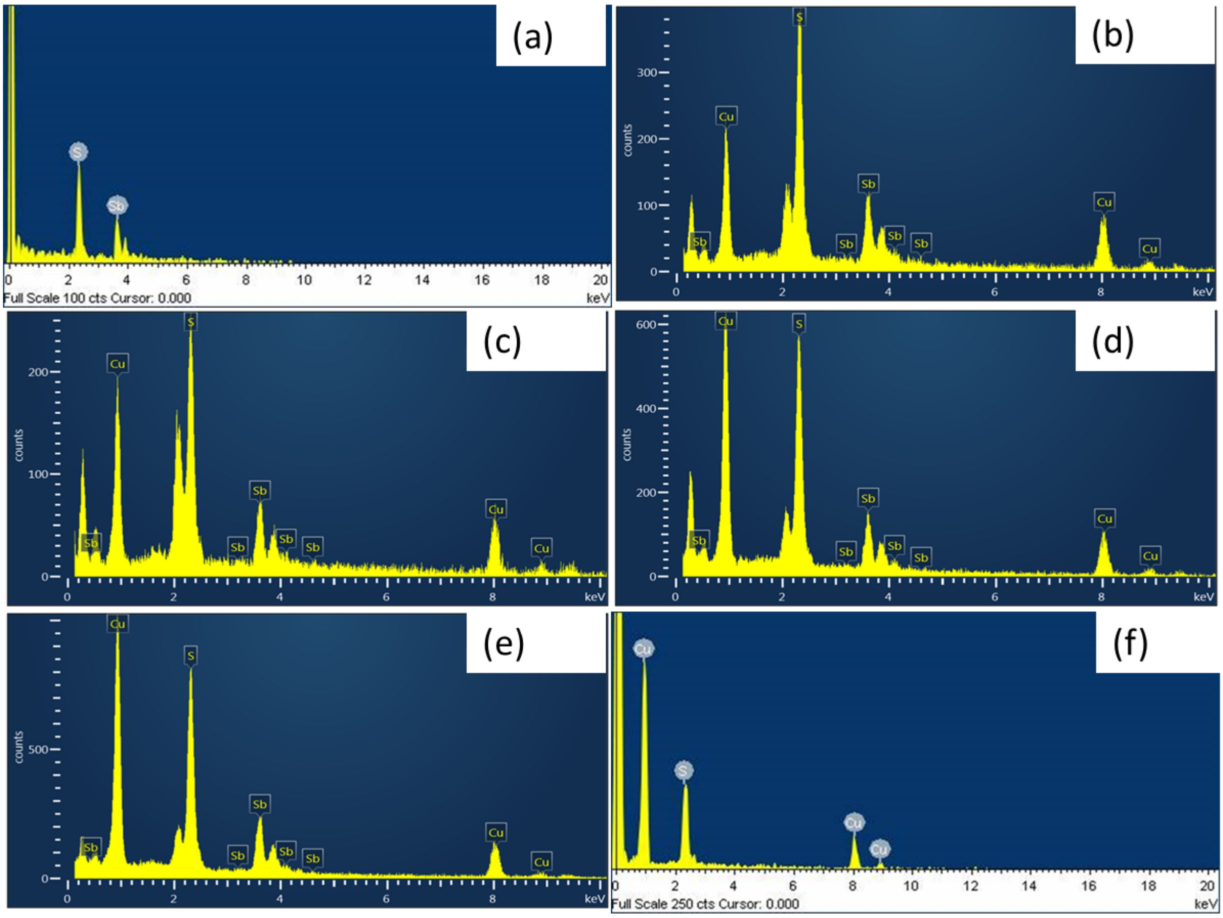


**Figure S4.** The EDX spectra of $Cu_{2x}Sb_{2(1-x)}S_y$ samples prepared by solventless thermolysis at 450 °C at various mole fractions where (a) x = 0, (b) x = 0.2, (c) x = 0.4, (d) x = 0.6, (e) x = 0.8 and (f) x = 1.

**Expected Cu and Sb content *vs* observed Cu and Sb content in the particulate ternary $Cu_{2x}Sb_{2(1-x)}S_y$ systems produced by solventless thermolysis**

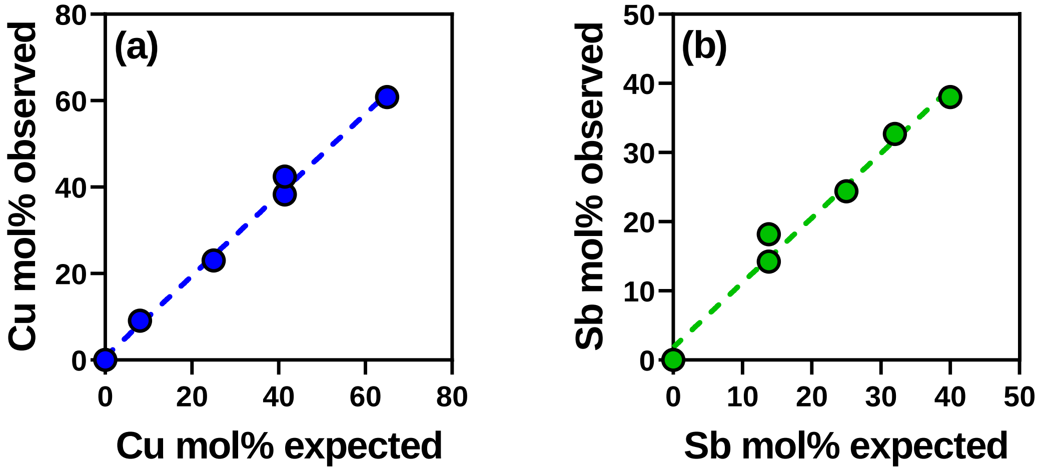


**Figure S5.** Linear relationship between the amount of copper expected and the amount of copper in the particulate $Cu_{2x}Sb_{2(1-x)}S_y$ systems synthesized by solventless thermolysis at 450 °C, obtained from EDX analysis.

**Table S2.** The content of Cu, Sb and S in $Cu_{2x}Sb_{2(1-x)}S_y$ prepared by solventless thermolysis at 450°C for 0 ≤ x ≤ 1, as calculated from the feed rate and found by EDX analysis.

| Mole Fraction | Elemental Composition Expected (Atomic %) | | | Stoichiometry expected | Elemental Composition Found by EDX (atomic %) | | | Stoichiometry Found By EDX |
|---|---|---|---|---|---|---|---|---|
| | Cu | Sb | S | | Cu | Sb | S | |
| 0 | 0 | 40 | 60 | $Sb_2S_3$ | 0 | 38.0 | 62.0 | $Sb_{1.9}S_{3.1}$ |
| 0.2 | 8 | 32 | 60 | $Cu_{0.4}Sb_{1.6}S_3$ | 9.1 | 32.7 | 58.2 | $Cu_{0.4}Sb_{1.6}S_{2.62}$ |
| 0.4 | 25 | 25 | 50 | $CuSbS_2$ | 23.03 | 24.41 | 52.56 | $Cu_{1.15}Sb_{1.2}S_{2.75}$ |
| 0.6 | 41.4 | 13.8 | 44.8 | $Cu_{12}Sb_4S_{13}$ | 38.27 | 18.18 | 43.55 | $Cu_{11.8}Sb_{5.63}S_{13.5}$ |
| 0.8 | 41.4 | 13.8 | 44.8 | $Cu_{12}Sb_4S_{13}$ | 42.41 | 14.22 | 43.37 | $Cu_{13.1}Sb_{4.40}S_{13.4}$ |
| 1 | 64.28 | 0 | 35.71 | $Cu_2S$ | 60.8 | 0 | 39.2 | $Cu_{1.82}S_{1.17}$ |

**Raman spectroscopy for particulate $Cu_{2x}Sb_{2(1-x)}S_y$ samples where x = 0, 0.2 and 1 from solventless thermolysis**

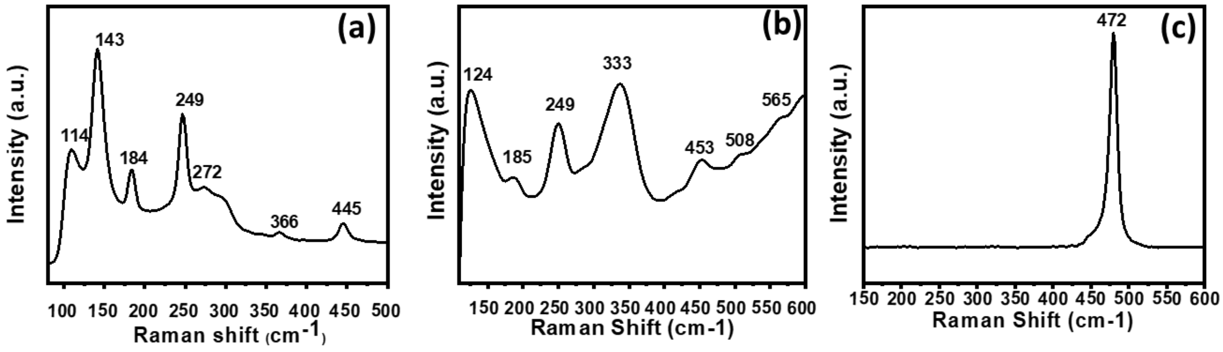


**Figure S6.** Raman spectra of the particulate $Cu_{2x}Sb_{2(1-x)}S_y$ systems where x = (a) 0, (b) 0.2 and (c)1.

## Photographs of the AACVD thin films of $Cu_{2x}Sb_{2(1-x)}S_y$

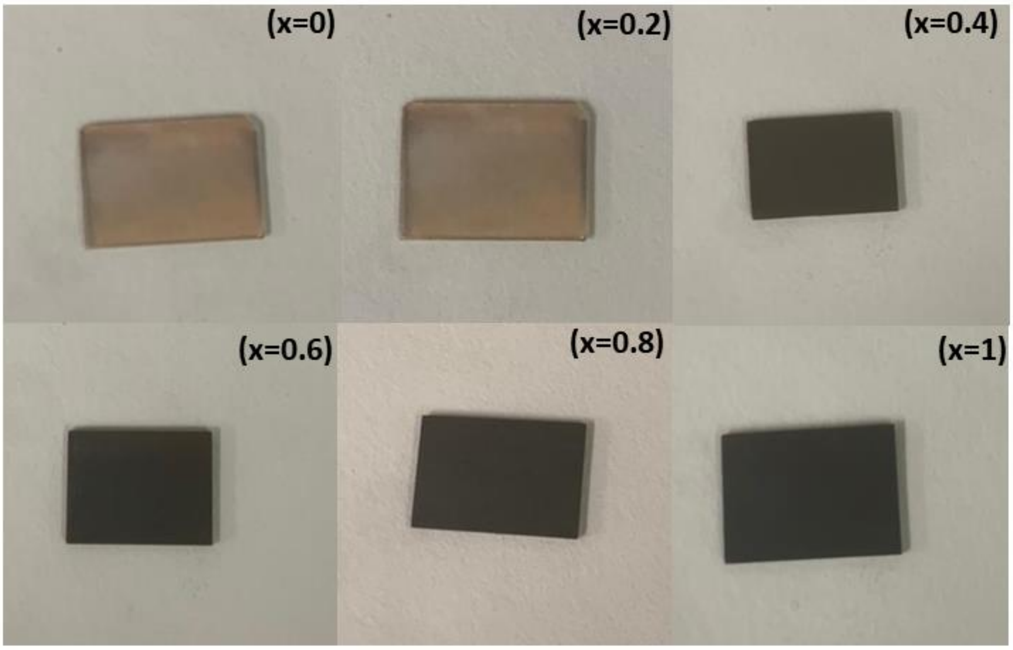


**Figure S7.** Figure showing photographs of the AACVD deposited thin films of $Cu_{2x}Sb_{2(1-x)}S_y$, where the particular x values are shown.

## Elemental analysis of the thin film $Cu_{2x}Sb_{2(1-x)}S_y$ samples produced by AACVD

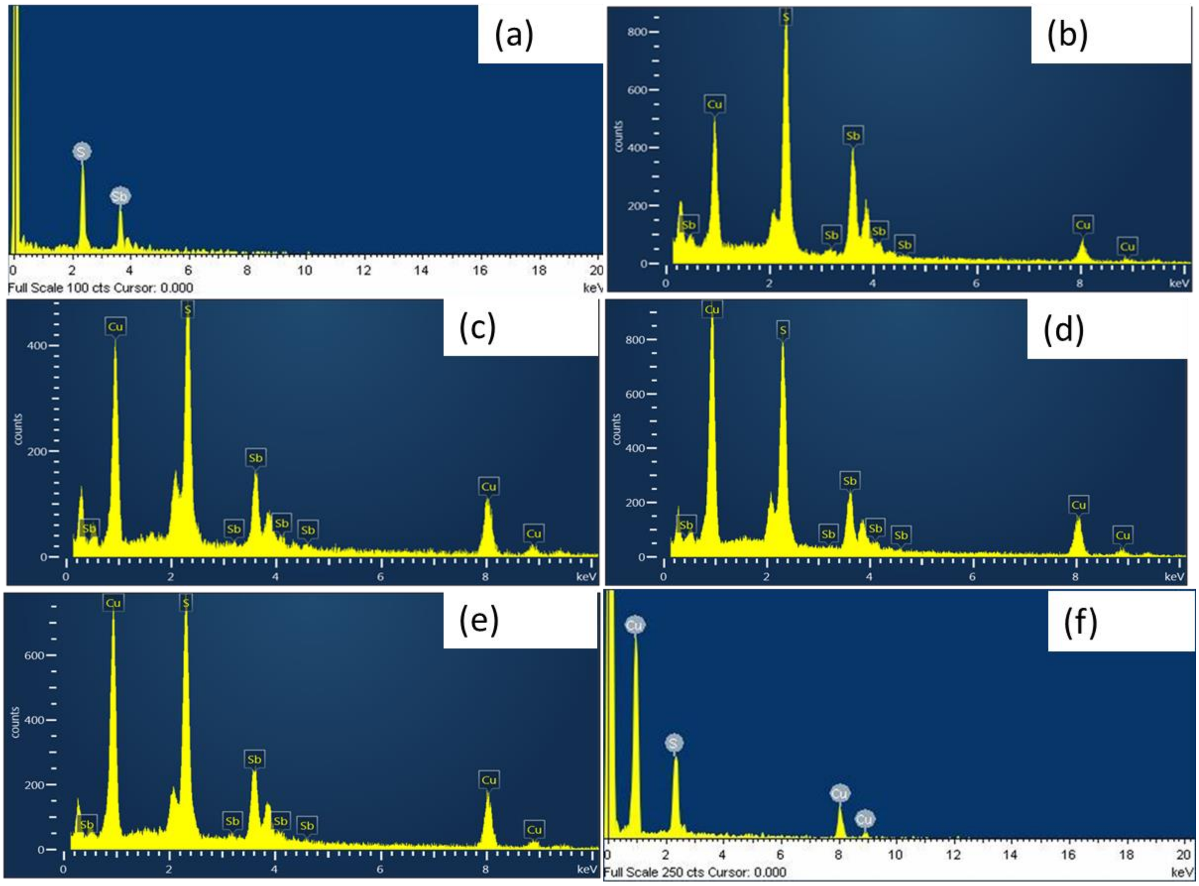

**Figure S8**. The EDX spectra of $Cu_{2x}Sb_{2(1-x)}S_y$ thin films prepared by AACVD at 450 °C at various Iron mole fraction (a) x = 0, (b) x = 0.2, (c) x = 0.4, (d) x = 0.6, (e) x = 0.8 and (f) x = 1.

**Expected Cu content *vs* observed Cu content in the ternary $Cu_{2x}Sb_{2(1-x)}S_y$ thin films deposited by AACVD**

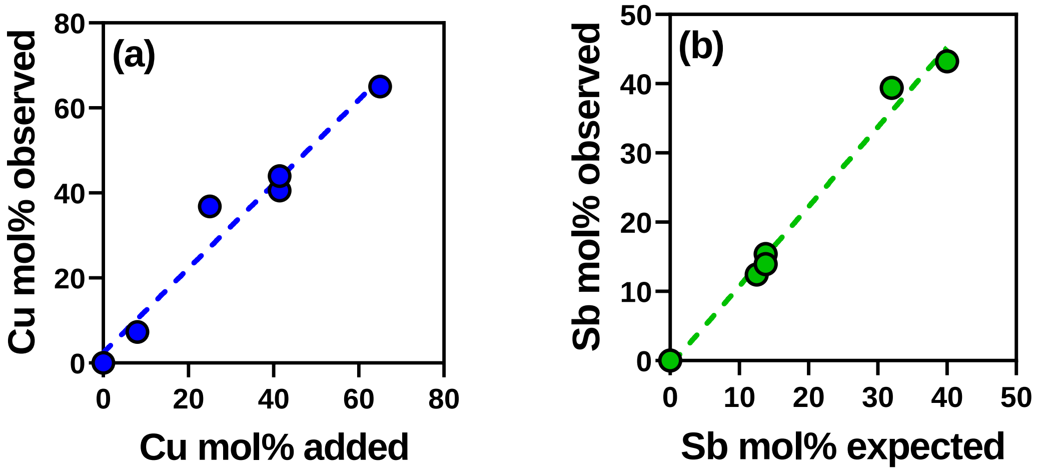


**Figure S9.** Linear relationship between the amount of copper expected and the amount of copper in $Cu_{2x}Sb_{2(1-x)}S_y$ thin films synthesized by the AACVD method at 450°C from EDX analysis.

**Table S3.** The content of Cu, Sb and S in $Cu_{2x}Sb_{2(1-x)}S_y$ thin film prepared by AACVD method at 450°C (x = 0, 0.2, 0.4, 0.6, 0.8 and 1 molar fraction of copper) calculated from the feed rate and found by EDX analysis.

| Mole Fraction | Elemental Composition Expected (Atomic %) | | | Stoichiometry expected | Elemental Composition Found by EDX (atomic %) | | | Stoichiometry Found By EDX |
|---|---|---|---|---|---|---|---|---|
| | Cu | Sb | S | | Cu | Sb | S | |
| 0 | 0 | 40 | 60 | $Sb_2S_3$ | 0 | 43.24 | 56.76 | $Sb_{2.16}S_{2.83}$ |
| 0.2 | 8 | 32 | 60 | $Cu_{0.4}Sb_{1.6}S_3$ | 7.30 | 39.38 | 53.32 | $Cu_{0.36}Sb_{1.96}S_{2.66}$ |
| 0.4 | 37.5 | 12.5 | 50 | $Cu_3SbS_4$ | 36.82 | 12.42 | 50.76 | $Cu_{2.94}Sb_{0.99}S_{4.06}$ |
| 0.6 | 41.4 | 13.8 | 44.8 | $Cu_{12}Sb_4S_{13}$ | 40.57 | 15.38 | 44.05 | $Cu_{11.76}Sb_{4.46}S_{12.77}$ |
| 0.8 | 41.4 | 13.8 | 44.8 | $Cu_{12}Sb_4S_{13}$ | 43.97 | 13.95 | 42.59 | $Cu_{12.75}Sb_{4.04}S_{12.35}$ |
| 1 | 66.65 | 0 | 33.35 | $Cu_2S$ | 65 | 0 | 35 | $Cu_{1.95}S_{1.05}$ |

**Raman spectra of thin film $Cu_{2x}Sb_{2(1-x)}S_y$ samples where x = 0, 0.2 and 1, deposited by AACVD.**

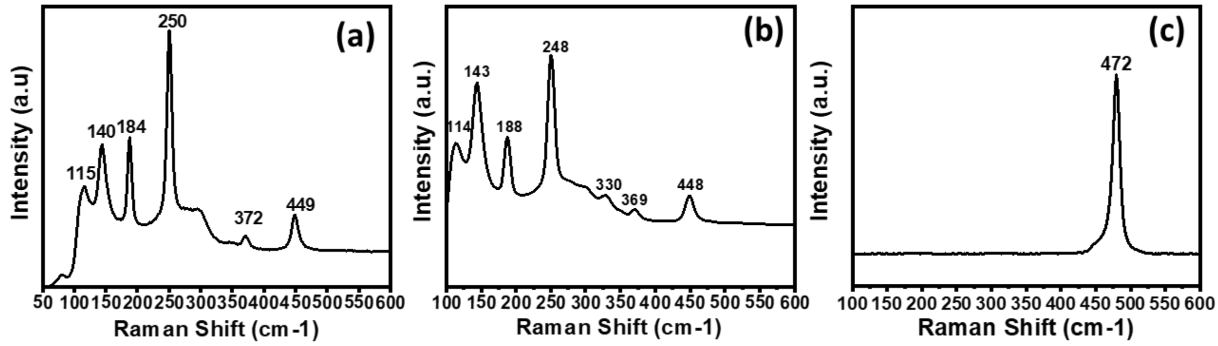


**Figure S10**. Raman spectra recorded for the AACVD-deposited thin films of $Cu_{2x}Sb_{2(1-x)}S_y$ where x = (a) 0 (b) 0.2 and (c) 1.

## References


1 I. Inagawa, S. Morimoto, T. Yamashita and I. Shirotani, Temperature Dependence of Transmission Loss of Chalcogenide Glass Fibers, *Jpn. J. Appl. Phys.*, 1997, **36**, 2229–2235.
